# Antidepressant–like effects of fish, krill oils and Vit B12 against exposure to stress environment in mice models: current status and pilot study

**DOI:** 10.1038/s41598-019-56360-8

**Published:** 2019-12-27

**Authors:** Parastoo Mojtahed Zadeh-Ardabili, Sima Kianpour Rad, Soheila Kianpour Rad, Abolfazl Movafagh

**Affiliations:** 1 Physiology Department, School of Medicine, Mashhad University of Medical Science, Mashhad, Iran; 2 Molecular Medicine Department, Faculty of Medicine, University of Malaya, 50603 Kuala Lumpur, Malaysia; 3 Faculty of Medicine, Qazvin University of Medical Science, Qazvin, Iran; 4 Department of Medical Genetics, School of Medicine, Shahid Beheshti University of Medical Sciences, Tehran, Iran

**Keywords:** Drug development, Cell type diversity, Depression, Neuroscience

## Abstract

Oxidative stress has significant role in pathophysiology of any kind of depression through actions of free radicals, non-radical molecules, and unbalancing antioxidant systems in body. In the current study, antidepressant responses of fish oil (FO), Neptune krill oil (NKO), vitamin B12 (Vit B12), and also imipramine (IMP) as the reference were studied. Natural light was employed to induce stress in the animals followed by oral administration of the drugs for 14 days. The antidepressant effect was assessed by tail suspension test (TST) and forced swimming test (FST), antioxidant enzymes and oxidative stress markers were then measured in the brain tissue of the animals. The administration of FO and NKO could significantly reduce the immobility of the animals; while, increasing climbing and swimming time compared to the normal saline in CUS-control group in TST and FST, similarly to IMP but not with Vit B12. Vit B12 could not effect on SOD activity and H_2_O_2_ level, but, cause decrease of the malondialdihydric (MDA) level and CAT activity, as well as increased the GPx and GSH activities. The rest treatments led to decrease of MDA, H_2_O_2_ levels and CAT activity and increase of GPx, SOD, GSH activities.

## Introduction

Depression is a chronic and devastating psychological complaint that millions of people suffer. Since 1950s up to the present time, antidepressants only have treated this disorder based on serendipitous alteration through the monoamine neurotransmitters, such as serotonin or noradrenaline. However, fundamental patho-physiological mechanisms of such illness are not fully understood^1,2^, but different pharmacologic therapeutics have been recently developed for managing depression^3^. It is demonstrated that administration of natural antidepressants in induced stress rodents are able to alleviate related symptoms^4,5^. Physicians are encouraged to search for natural compounds as effective drugs due to their lower side effects compared to the chemical drugs. For instance, recent findings show that omega-3 fatty acids are able to improve depression and anxiety, effectively without having serious adverse effects^6^. In addition, it is well understood that n-3 polyunsaturated fatty acids (n-3 PUFAs) which include eicosapentaenoic acid (EPA) and docosahexaenoic acid (DHA), possess some bioactivity effects, such as, anti-inflammatory, anti-nociceptive, antioxidant, anti-depressant, anti-anxiety, anti-cardiovascular, etc.^7,8^. For instance, brain is made up to 60% of different lipids which are mostly made of PUFAs, particularly, DHA^9^ which contribute to healthy brain and improve variety of brain functions, such as memory and learning^10,11^. Bloch and Hannestad^12^ demonstrated that EPA and DHA contain only a little or even no-significant antidepressant effects, without having any noticeable differences. However, Sublette *et al*.^13^ suggested that only those supplements which consist of EPA ≥ 60% of the total EPA and DHA possess an effective anti-mild-depressant activity. It is reported that either fish oil or krill oil which is extracted from small shrimplike planktonic crustacean of open seas, possess high level of essential polyunsaturated lipids, EPA and DHA^14^. In addition of fatty acids, some vitamins have shown to possess mild antidepressant effect and are prescribed by physicians to improve patient’s depression and anxiety. As an example, administration of Vit B12 as an exogenous antioxidant at dose of 1 mg/daily to those patients suffering from depression [20] can improve variety of symptoms of mental and depression disorders^15^.

To study effectiveness of drugs as antidepressant in rodents, some distinctive and clear depressive like behaviours before and after treatments have been suggested to be assessed with some reliable tests, such as tails suspension (TST) and forced swimming tests (FST). In FST, novel antidepressant drugs in comparison with the control group could reduce immobility in animals. Also, some other behaviours, such as swimming and climbing are strongly suggested to be scored separately^16^. Swimming time is increased by selective serotonin reuptake inhibitors, on the contrary, climbing is motivated by selective norepinephrine reuptake inhibitors. In both tests, the immobility time of animals is related to the state of depression which is expected to decrease by using effective antidepressants^17^.

The role of ROS, such as H_2_O_2_ or MDA which are formed from lipid peroxidation of polyunsaturated fatty acids in depression is strongly highlighted^18–20^ and show to associate with lowering antioxidant defence or failure to repair oxidative damage. In order to eliminate the harmful effects of oxidative stress, body can get benefit from both endogenous and exogenous antioxidant systems^21,22^. Studies show that antidepressants affect antioxidant systems in brain to subside of free radicals to alleviate the symptoms of such disorders^23,24^.

In the present novel work, antidepressive-like-effects are evaluated in four different groups of animals which are treated by IMP, FO, NKO, Vit B12 to find their different actions against depression. Also, SOD, CAT, GPX, and GSH activity (major endogenous antioxidant enzymes) and also MDA and H_2_O_2_ level (oxidative stress markers) of the hippocampal of the experimental animals are assessed. Also the effect of the compounds on the enzymatic systems is evaluated to find out their connection to antidepressant-like effect. Furthermore, the aforementioned activities of FO and NKO are compared together and to a moderate natural antidepressant (Vit B12) and also to imipramine as a strong chemical antidepressant.

## Results

### Effect of the treatments on the immobility time in TST

**Figure 1:**
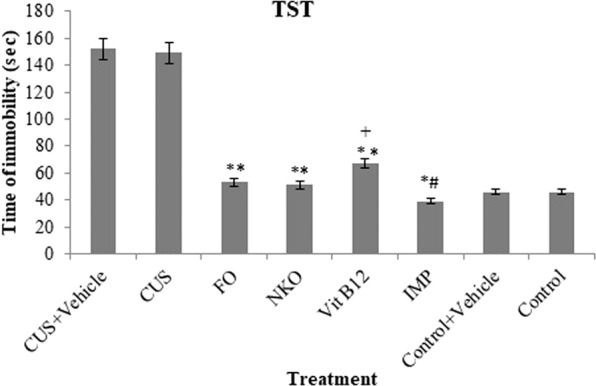
Effects of drugs on immobility time in TST. All animals (n = 90), except the control groups (n = 18) receive CUS. CUS (n = 9) and control (n = 9) animals do not receive any drugs or vehicle and the rests are orally administered with vehicle and the treatments. The treated groups (n = 72) are treated with FO (0.32 g/kg/day, n = 18), NKO (0.5 g/kg/day, n = 18), Vit B12 (1 mg/kg/day, n = 18) and IMP (5 mg/kg/day, n = 18). Results are expressed as mean ± SEM of immobility time (in seconds). Differences are analysed using two-way ANOVA followed by post hoc Dunnett’s test. For statistical significance, *p < 0.05 and **p < 0.01 when compared to the CUS group and ^+^p < 0.05 when FO and NKO compared to Vit B12. ^ǂ^When compared to the control. FO: Fish oil; NKO: Neptune Krill Oil; CUS: Chronic unpredictable stress; IMP: Imipramine EPA: Eicosapentaenoic Acid; DHA: Docosahexaenoic Acid; PLs: Phospholipids; TGA: Triglycerides; ANOVA: analyses of variance; SEM: standard error of the mean. Vehicle = 7% Tween 80 in normal saline.

Effect on the immobility time by the oral daily treatments is exhibited in Fig. 1. Analysis showed that FO, NKO, Vit B12 and IMP could significantly decrease the time of immobility compared to the CUS group with no significant difference with control group. There was no noticeable difference between NKO, FO, and Vit B12 and also noticed that IMP was more effective to increase the immobility time than the other three supplements.

### Effect of the treatments on the depressive-like behaviours in FST

**Figure 2:**
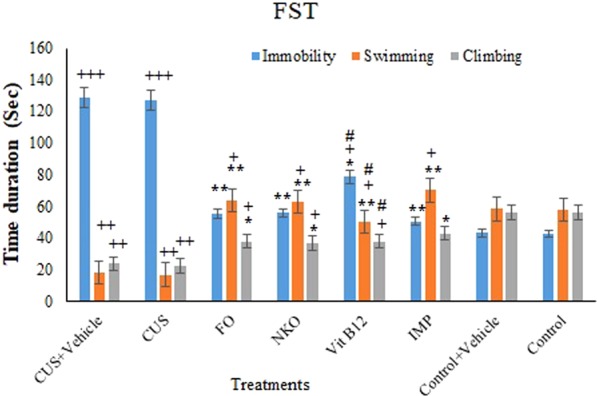
Effects of drugs on immobility, swimming, climbing time in FST. All animals (n = 90), except the control groups (n = 18) receive CUS. CUS (n = 9) and control (n = 9) animals do not receive any drugs or vehicle and the rests are orally administered with vehicle and the treatments. The treated groups (n = 72) are administrated with FO (0.32 g/kg/day, n = 18), NKO (0.5 g/kg/day, n = 18), Vit B12 (1 mg/kg/day, n = 18) and IMP (5 mg/kg/day, n = 18). Results are expressed as mean ± SEM. The differences are analysed using two-way ANOVA followed by a post hoc Dunnett’s test. For statistical significance, *p < 0.05, **p < 0.01, and ***p < 0.001 when compared to the CUS group ^+^p < 0.05 when compared to the control and ^ǂ^p < 0.05 when FO and NKO compared to Vit B12. FO:Fish oil; NKO: Neptune Krill Oil; CUS: Chronic unpredictable stress; IMP: Imipramine FST: Fast Swimming Test; CUS: Chronic unpredictable stress; ANOVA: analyses of variance; SEM: standard error of the mean. Vehicle = 7% Tween 80 in normal saline.

Figure 2 depicts antidepressant activity of the treatments using FST with analysing the time duration of the immobility, swimming and climbing of the animals. Post hoc analysis showed that FO, NKO, and IMP could significantly reduce the immobility time; while increasing the climbing and swimming times compared to the CUS animals. But, Vit B12 was found to decrease the immobility time and increase the swimming time without any significant effect on the climbing. Also, no significant difference was observed between FO, NKO, and IMP in changing immobility, swimming, and climbing times and found to be more sensitive than Vit B12 (P < 0.05). There were no significant difference between climbing, swimming and immobility times of those animals treated with FO, NKO, IMP and control group.

### Descriptive analysis of depressive-like behaviours in FST and re-test (frequency, duration, and latency) in CUS and control animals

Table 1 lists the frequency, duration, and latency of depressive-like behaviours in FST. Swimming in both latency and duration was detected as the longest behaviour, followed by immobility; however climbing was noticed as the shortest behaviour. In fact, in both depression induced animals (CUS) and undepressed group (control), the priority was climbing followed by swimming and finally immobility.

**Table 1:** Overall latency, frequency and duration of behaviours recorded by the stressed (CUS) and un-stressed (control) animals during FST and re-test.

	Latency(s)		Frequency		Duration(s)	
Climbing	Test	Re-test	Test	Re-test	Test	Re-test
CUS + Vehicle	17.28 ± 2.0*	16.29 ± 1.1*	6.88 ± 1.3*	5.81 ± 1.8*	69.26 ± 1.1**	71.63 ± 3.5**
Control + Vehicle	10.1 ± 0.2	9.7 ± 0.8	8.2 ± 1.1	9 ± 0.1	28.18 ± 1.4	25.55 ± 4.2
Swimming	Test	Re-test	Test	Re-test	Test	Re-test
CUS + Vehicle	22.66 ± 3.1*	24.27 ± 5.1*	11.11 ± 1.7*	9.98 ± 2.3*	150.12 ± 4.1**	147.01 ± 7.3**
Control + Vehicle	16.02 ± 2.1	18.18 ± 0.6	13.66 ± 2.0	15.3 ± 1.7	92.98 ± 0.14	92.18 ± 3.2
Immobility	Test	Re-test	Test	Re-test	Test	Re-test
CUS + Vehicle	71.1 ± 1**	69.09 ± 9**	8.04 ± 2.1*	8.91 ± 3.5**	144.8 ± 5.1**	143.34 ± 10.01**
Control + Vehicle	112.2 ± 0.3	110 ± 3.1	4.08 ± 1.1	3.5 ± 0.01	51.18 ± 3.5	48.9 ± 5.5

Values ± SEM, number of cases = 18 in each groups of CUS and control mice in 6 min of test and repeated-FTS.

*Significantly different from stressed mice (CUS) and control group, *p* < 0.05 (ANOVA followed by Duncan’s test). ANOVA = analyses of variance; SEM = Standard Error of Mean; CUS = Chronic Unpredictable Stress; FTS: Forced Swimming Test.

Vehicle = 7% Tween 80 in normal saline.

### Analysis of frequency of depressive like behaviours in the treated and untreated animals in FTS in 6 min with 3 intervals

**Figure 3:**
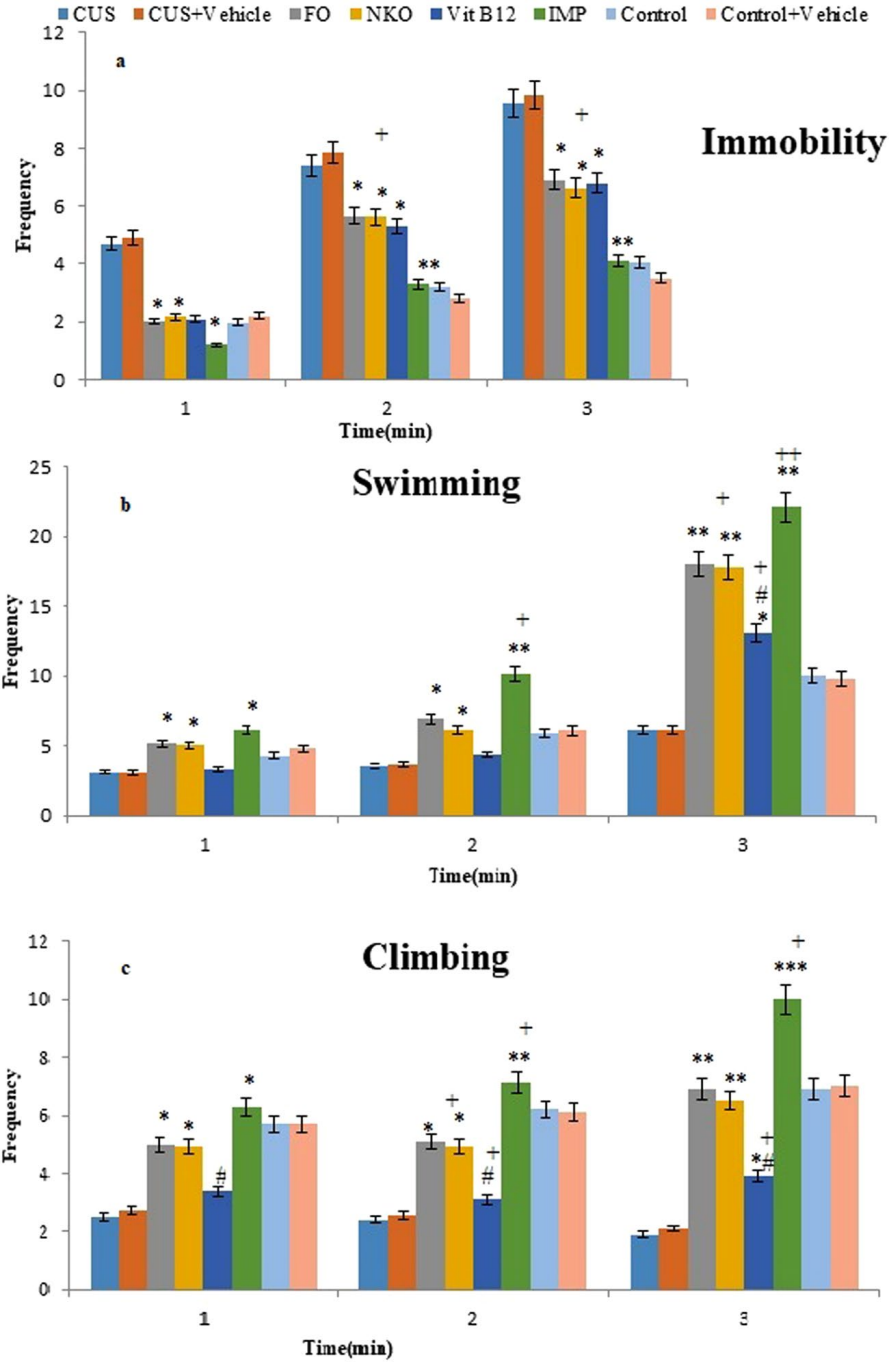
Frequency of behaviours, immobility (**a**), swimming (**b**), and climbing (**c**) are recorded during retest 1, of repeated FST during 6 min (every 2 min). All animals (n = 90), except the control groups (n = 18) receive CUS. CUS (n = 9) and control (n = 9) animals do not receive any drugs or vehicle and the rests are orally administered with vehicle and the treatments. The treated groups (n = 72) are administrated with FO (0.32 g/kg/day, n = 18), NKO (0.5 g/kg/day, n = 18), Vit B12 (1 mg/kg/day, n = 18) and IMP (5 mg/kg/day, n = 18). Results are expressed as mean ± SEM. The differences are analysed using two-way ANOVA followed by a post hoc Dunnett’s test. For statistical significance, *p < 0.05 and **p < 0.01 when compared to the CUS group and ^ǂ^p < 0.05 when FO and NKO compared to Vit B12 and ^+^p < 0.05 when compared to the control. FO:Fish oil; NKO: Neptune Krill Oil; IMP: Imipramine; CUS: Chronic unpredictable stress; FST: Fast Swimming Test. Vehicle = 7% Tween 80 in normal saline; ANOVA: analyses of variance; SEM: standard error of the mean.

The frequency of immobility, swimming, and climbing in 6 min with 2 mins interval is depicted in Fig. 3. Post hoc analysis showed (Fig. 3a) that frequency of the last interval analysis was significantly decreased in those mice which were treated with FO, NKO, Vit B12 (p < 0.05) and IMP (p < 0.01), when compared to the CUS group. No significant difference was detected among the two first treatments at each time in the frequency of immobility; while, IMP showed the highest effect on reducing the frequency of immobility. Figure 3b depicts that the treatments could significantly enhance frequency of swimming compared to vehicle in CUS animals at each interval, but, both FO and NKO enhanced frequency more than Vit B12 (p < 0.05). Figure 3c shows that IMP, FO, NKO could increase climbing frequency at each interval significantly more than vehicle in CUS group (p < 0.001, p < 0.01); while, no drastic difference was noticed in the group treated with Vit B12 and also CUS animals.

### Antioxidant enzymes profile in brain tissue

**Figure 4:**
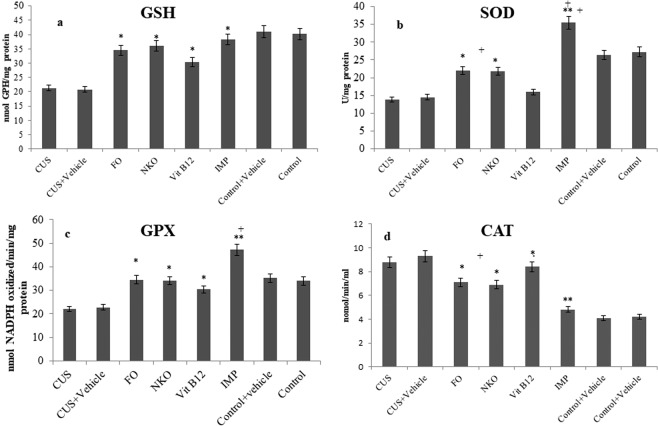
Effects of drugs on antioxidant enzyme activity. All animals (n = 90), except the control groups (n = 18) receive CUS. CUS (n = 9) and control (n = 9) animals do not receive any drugs or vehicle and the rests are orally administered with vehicle and the treatments. The treated groups (n = 72) are administrated with FO (0.32 g/kg/day, n = 18), NKO (0.5 g/kg/day, n = 18), Vit B12 (1 mg/kg/day, n = 18) and IMP (5 mg/kg/day, n = 18). The hippocampi of the animals are analysed for GSH (**a**), SOD (**b**), GPX (**c**) and CAT (**d**) activity. Results are expressed as mean ± SEM. The differences are analysed using two-way ANOVA followed by post hoc Dunnett’s test. For statistical significance, *p < 0.05, **p < 0.01, and ***p < 0.001 when compared to the CUS group and ^ǂ^p < 0.05 when FO and NKO compared to Vit B12 and ^+^p < 0.05 when compared to the control. FO: Fish oil; NKO: Neptune Krill Oil; CUS: Chronic unpredictable stress; IMP: Imipramine; SOD: Superoxide dismutase; GSH: Glutathione; GPX: Glutathione Peroxidase; CAT: Catalase; ANOVA: analyses of variance; SEM: standard error of the mean. Vehicle = 7% Tween 80 in normal saline.

As depicted in Fig. 4(a–c), except Vit B12 which could not drastically change the SOD activity compared to the control group, the rest treatments, significantly increase the activity of GSH, SOD, and GPX in brain tissue compared to the CUS animals. Both NKO and FO were found to be more effective in changing GPX enzyme activities than Vit B12 (*p* < 0.05). Figure 4(b) shows that all the treatments could decrease CAT activity compared to CUS animals (*p* < 0.05) with no significant difference between FO and NKO, while the two oily compounds were found to be more effective than Vit B12 (*p* < 0.05). All the treatments could restore the GSH activity of the stressed animals to that of the unstressed group. IMP increased SOD activity higher than that of unstressed animals (control), however SOD activity in control animals was higher than that of FO, NKO and Vit B12 treated animals. There was no drastic difference of GPx activity in NKO, FO, Vit B12 treated animals and control group, however, IMP could increase GPX activity more than that of control animals. Among the treatment, only IMP could restore the CAT activity to that of control animals.

### Protective effect of treatments against MDA and H_2_O_2_

**Figure 5:**
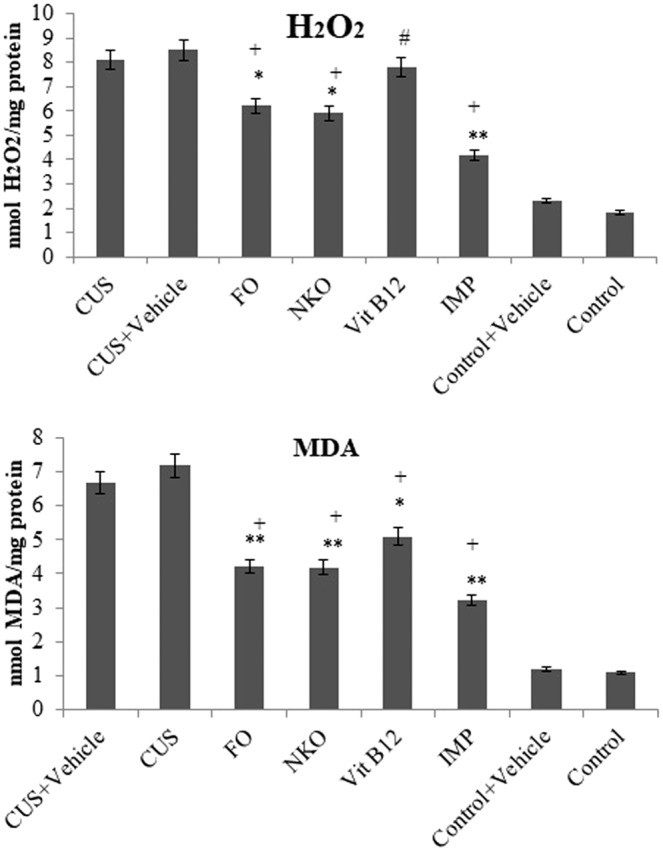
Effects of drugs on antioxidant enzyme activity. All animals (n = 90), except the control groups (n = 18) receive CUS. CUS (n = 9) and control (n = 9) animals do not receive any drugs or vehicle and the rests are orally administered with vehicle and the treatments. The treated groups (n = 72) are administrated with FO (0.32 g/kg/day, n = 18), NKO (0.5 g/kg/day, n = 18), Vit B12 (1 mg/kg/day, n = 18) and IMP (5 mg/kg/day, n = 18). The hippocampi of the animals are analysed for MDA (a), H2O2(b), levels. Results are expressed as mean ± Standard Error of Mean (SEM). The differences were analysed using two-way ANOVA followed by a post hoc Dunnett’s test. For statistical significance, *p < 0.05, **p < 0.01, and ***p < 0.001 when compared to the CUS group and ^ǂ^p < 0.05 when FO and NKO compared to Vit B12 and ^+^p < 0.05 when compared to the control. FO:Fish oil; NKO: Neptune Krill Oil; CUS: Chronic unpredictable stress; IMP: Imipramine; MDA: Malondialdehyde; ANOVA: analyses of variance; SEM: standard error of the mean. Vehicle = 7% Tween 80 in normal saline.

Those mice which were exposed to stress showed increase in their hippocampal MDA and H_2_O_2_ levels compared to CUS animals. As it is noticed in Fig. 5, the oily supplements with the same effect could lower both free radical levels (p < 0.05), but Vit B12 could significantly decrease the MDA with no change in H_2_O_2_ level compared to CUS group. Significant differences were found in H_2_O_2_ and MDA levels in all the treated groups and those of control animals.

## Discussion

The treated animals with FO (0.32 g/kg/day) and NKO (0.5 g/kg/day), which reached the balanced value of their EPA and DHA contents (DHA*/*EPA*)* were found to be equally effective against depression. This activity was found through chronic mouse behaviourally models of despair tasks (TST and FST), which are well-known as screening paradigms to study new antidepressants in term of their efficacy in rodent models. CUS-induced mice model was successfully a simulation for depressive status by increasing time of immobility in both FST and TST, and also by decreasing swimming and climbing times in FST. Such changes were also accompanied by reduction of GSH, SOD, and GPX and increasing of CAT activities as well as rising MDA and H_2_O_2_ levels in brain tissue. Moreover, the finding suggested that Vit B12 at 1 mg/kg/day possessed lower effect in changing antioxidant enzymes activity in CUS animals and acted differently having lower effect against depressive symptoms in comparison with NKO and FO. On the contrary, IMP at 5 mg/kg/day having highest effect on reversing those antioxidant enzymes activities as well as reduction of ROS level in CUS animals in comparison with the control animals could show highest effect against depression signs in the mice. Therefore, the antidepressant like effect of the treatments on CUS-induced mice might be related to the alterations of antioxidant defence.

It was demonstrated that in FST and TST, duration, latency and frequency of immobility can be the result of an inability or reluctance to keep endeavour rather than a generalized hypoactivity^25^ in animals. For instance, climbing or swimming as active behaviours normally help animals to escape and relieve their stress, while immobility, a passive behaviour, may be a safer way to preserve energy and waiting for a possible escape. It is interesting to note that animal’s choice of behaviours are different and depends on multiple factors, such as pre-exposure, energy status and type of treatment. For example, antidepressants are found to postpone the transition from active to passive coping strategies; however, factors that are noticed to be associated with depression can accelerate such transition^16^. There are always some pitfalls during FST which should be minimalized as possible. It is important to know that whether treatments could help animals learn some depressive like behaviours as the strategy against depression or not. Moreover, some other factors, such as furs effect (trapping air between furs which leads to buoyancy) in rodents^26^ when they are immobile should be regarded. Therefore, to minimize the potential biases, the frequency, latency and finally duration of behaviours in animals in control, stressed and treated groups in FST should be compared^26^. As listed in Table 1, climbing was the first behaviour emerged in both unstressed and stressed mice, followed by swimming as the next priority and then the animals started to be immobile. The results revealed that swimming, climbing and immobility in the tested animals were considered as internal reaction without any effect of external factors, such as the treatments. In addition to above reasons, the significant differences found between the stressed and unstressed mice in the descriptive analysis of depressive-like behaviours could be regarded as double confirmation of successful induction of chronic stress in mice.

According to the previous study which suggests evaluation of frequency of each depressive like behaviour to achieve the correct analysis^27^, the frequency of each behaviour in the repeated FST for 6 min with 3 intervals (each was 2 min) was assessed. The last interval was considered as the main important time to find out and compare the three behaviours in the treated animals. The swimming as the longest behaviour possessed the highest frequency than immobility and climbing in the treated mice with FO, NKO, as well as Vit B12 and IMP. It is demonstrate that those antidepressants which could increase serotonergic neurotransmission cause longer swimming duration, whereas those which are able to increase catacholaminergic neurotransmission make longer struggling durations just as climbing^16^. In the current study, all the treatments may show antidepressant activity through increasing serotonergic neurotransmission. In addition, the results of FST and TST clearly indicated that decreasing the time of immobility could be resulted from increasing both climbing and swimming times with oral administration of NKO as same as FO and IMP. However, Vit B12 could only reduce immobility time by elevating swimming with no drastic effect on climbing time and frequency in the stressed mice. This suggest that FO and NKO act similarly to IMP which are considered as norepinephrinergic drugs with mixed serotonin noradrenaline reuptake inhibitor^28^. However, the two oily compounds were more effective with different action as compared to Vit B12 which is known as serotonin reuptake blockers^29^.

It is demonstrated that anti-psychotics which are clinically effective antidepressants possess anti-immobility effects in either TST or FST via stimulating antioxidant enzymes activity and depleting ROS, accordingly^30^. Brain works with complex antioxidant mechanism and therefor is able to neutralize the harmful effects of ROS. However, in depression, the deficiency of antioxidant mechanisms and also changes in pro-inflammatory cytokine system cause elevation in free radical formation^31^. Antidepressants can raise brain’s serotonin level and accompanies improvement and increases several antioxidant enzymes which are responsible for protecting lipid from oxidation^32^. This increase of antioxidant system activity by antidepressant could lead depletion of ROSs and maybe some natural compounds with antidepressant effect possess antioxidant properties and decrease the level of ROS^33^. Our study suggests that FO and NKO having equal effect similar to other treatments (IMP and Vit B12) can increase activity of GSH, GPX, and SOD in CUS group; however, all treatments retarded CAT activity, but Vit B12 cannot increase SOD function. There was no difference between FO, NKO and Vit B12 only in reducing H_2_O_2_ level, suggesting that the two oily compounds were more successful in reducing ROS than Vit B12. There was a positive correlation between immobility time (Index of depression in animals) and the two measured markers of oxidative stress (MDA, H_2_O_2_) in CUS-treated animals. This suggests that defensive action against oxidative stress might be due to possible mechanisms of antidepressant-like effect of the treatments. Further detailed studies are definitely required to explore the exact possible mechanisms. Also, the immobility duration affected with treatments measured by both FST and TST correlated negatively with activities of SOD, GPX, GSH and positively with CAT activity. It is found that antidepressant-like effects of treatments were related to defensive action against oxidative stress. Nevertheless, another possibility for such effect is that increasing the scavenging activity of GPX, SOD, and GSH, can lower ROS levels. Although CAT is known to have low activity in brain, but it becomes more crucial in removing H_2_O_2_ compared to GPX and hence the levels of catalase for scavenging of H_2_O_2_ is higher than GPX in brain^34^. Therefore, it is hypothesized that increasing of GPX, SOD activities and also antioxidant properties of treatments can decrease H_2_O_2_ level in brain of the stressed mice which led decreasing CAT activity. Different anti-depressant effects among treatments might be due to different levels in changing SOD, CAT, and GPx activities.

## Conclusions

Although there are differences in structure of NKO and FO, but they show exactly the same antidepressant activity with the same effect on antioxidant enzymes. This can be due to the balance of EPA/DHA, explaining the fact that EPA and DHA in the two oily compounds are the most possible factors for such bioactivity effects.

Dissimilar effects between FO, NKO and Vit B12 can be due to different cascades they can change. However, it is found that FO and NKO are more effective than Vit B12 against depression in mice, but it cannot be suggested that the oily compounds have priority to be used as possible antidepressant. This is due to their different application in suppressing depression symptoms similar to TCA and SSRI.

## Methods and Materials

### Animals and experimental set up

One-hundred eight adult male Swiss albino mice (20–25 g) were obtained from the Institute of Experimental Animals (Kuala Lumpur, Malaysia) and manipulated in this study. They were kept in plastic cages with dimension of 41 × 34 × 16 cm (each cages included 10 mice) under optimum environment (23 ± 2 °C, 12 h natural 12 h light/ 12 h dark cycle) having free access to water, *libitum* and standard chow pellets as a standard basal diet. The animals were acclimatized to laboratory conditions without having any access to food for 1 day before doing the experiments. The whole experimental processes, including animal work in this study were approved by the Animal Care Committee of the Faculty of Medicine’s Animal House, Institutional Animal Care and Use Committee; University Malaya (UM), according to the guideline and policy of University Malaya; regarding the care and use of animals for scientific purposes with the reference number animals (Ethic no. 2015-09-11/BMS/R/MAA).

### Chronic unpredictable stress paradigm overnight (CUS)

Light was used as stress inducer for mice and the schedule of using overnight illumination was 10 W LED, 15 Hz for 12 hours which the mice received it once per day for 3 weeks consecutively^35^.

### Drug preparation

IMP (99%) (Merck, Darmstadt, Germany) and also Vit B12 (Merck, Darmstadt, Germany) were dissolved in water to prepare a dose of 5 mg/kg/day which was suggested to use with minimum antidepressant effect in rodent^36^ and 1 mg/kg/day, respectively for administrating orally to the animals (based on the AIN-76 diets formulation)^37^. The calculation of the concentration of diets for the oily supplements were based on both AIN-93G^38^ and literature and for preparing the calculated doses, they were dissolved in 7% Tween 80 in normal saline^39^. The oily treatments were included: fish oil (500 mg) (*Nature Made*® Fish oil, California, USA), Neptune brand krill oil (500 mg) (*NKO®*, Neptune Technology & Bioressources Inc (NEPT), and Darmstadt, Germany), and containing substantially high level of non-ether phospholipids, ether phospholipids, and astaxanthin. Because it is important to administer exactly the same doses of the treatments, the two oily drugs were balanced for their content of DHA and EPA, that is, the average dose of 0.5 g/kg of NKO and 0.32 g/kg of FO were used in the established program. Products representative of the NKO and FO manufacturers were selected under omega-3 oils category in Malaysia local production. All the doses were chosen based on earlier reports that, the two oily treatments, Vit B12, and IMP were effective in producing physiological and behavioural effects in rodents^29^. The diets were stored in vacuum bags to prevent n-3 PUFA oxidation. Full details of the selected products and batch numbers are listed in Table 2.

**Table 2:** Contents of each pack of Neptune Krill (NKO) oil and Fish oil (FO) which are used in the study.

Supplement Fact	Neptune Krill oil (NKO™), 500 mg Patch NO: 060822	Fish oil (Nature’s Made ^®^), 500 mg Patch NO: 031604026622
Omega-3 fatty Acid, mg	115 (23 g/100 g)	—
EPA, mg	60 (12 g/100 g)	90 (12 g/100 g)
DHA, mg	35(7 g/100 g)	60 (8 g/100 g)
PLs, mg	195(39 g/100 g)	—
TGA, mg	—	150 (18.75 g/100 g)
Esterified Astaxanthin, mcg	375(0.075 g/100 g)	—

FO = Fish oil; NKO = Neptune Krill oil; EPA: eicosapentaenoic acid; DHA: docosahexaenoic acid; PLs: Phospholipids; TGA: Triglyceride.

### Animal groupings

**Figure 6:**
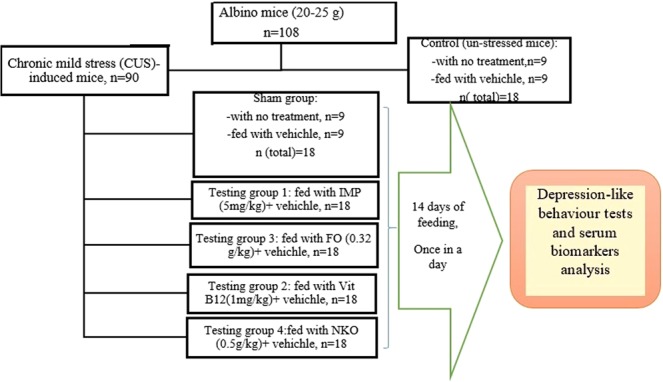
Flowchart of study design and number of mice which were used in each stage. All animals (n = 90), except the control groups (n = 18) receive CUS. CUS (n = 9) and control (n = 9) animals do not receive any drugs or vehicle and the rests are orally administered with vehicle and the treatments. The treated groups (n = 72) are administrated with FO (0.32 g/kg/day, n = 18), NKO (0.5 g/kg/day, n = 18), Vit B12 (1 mg/kg/day, n = 18) and IMP (5 mg/kg/day, n = 18). FO: Fish oil; NKO: Neptune Krill Oil; IMP: Imipramine; CUS: Chronic unpredictable stress. Vehicle = 7% Tween 80 in normal saline.

Figure 6 shows that except 18 mice (the control group), the rests were under chronic mild stress (CUS). In the CUS experiment, the mice (n = 90) were randomly divided into five main groups (n = 18 per group) as follow:

G1: Subgroup 1, normal control group fed on basal diet and administrated orally with 7% Tween 80 in normal saline (vehicle), n = 9, Subgroup 2: normal control group administrated with neither vehicle nor drugs (n = 9).

G2 (Sham): Subgroup 1, CUS mice + Vehicle (n = 9), Subgroup 2: CUS mice administrated with neither vehicle nor drugs (n = 9).

G3: CUS mice + IMP (5 mg/kg) + Vehicle

G4: CUS mice + Vit B12 (1 mg/kg) + Vehicle

G5: CUS mice + FO (0.32 g/kg) + Vehicle

G6: CUS + NKO (0.5 g/kg) + Vehicle

The concentration used for each drugs were based on their effectiveness which were found in the literature. First, the two oily drugs were diluted in 7% Tween 80 in normal saline for reaching the balanced of EPA and DHA and the control and CUS groups received same volume of saline containing 7% Tween^40^.

### Drug administration and

All the drugs including, FO, NKO, Vit B12 and IMP and also saline were given at a volume of 5 ml/kg once per day for 14 days starting, 1 week after the beginning of the CUS procedure^40^.

The first session of FST (pre-session) was done on the day after the last stress period, and the second on the following day. Then 1 day later, the mice were tested for TST and after 24 hours, all the animals left without any treatment until the following morning for re-FST. Then the all animals were sacrificed by the decapitation under general anesthesia using 2% isoflurane (Merck, Darmstadt, Germany). The hippocampi of the animals were detached and homogenized (1:10 w/v) in the HEPES buffer (20 mM, pH 7.0) for assessment of the antioxidant enzymes activity and ROS levels.

**Figure 7:**
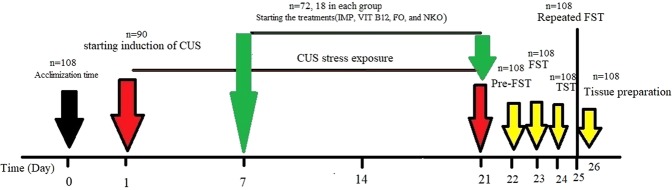
Timeline of study design used to study antidepressant–like effects of fish, krill oils, Vit B12 and also IMP against exposure to stress environment in mice. All animals (n = 90), except the control groups (n = 18) receive CUS. CUS (n = 9) and control (n = 9) animals do not receive any drugs or vehicle and the rests are orally administered with vehicle and the treatments. The treated groups (n = 72) are administrated with FO (0.32 g/kg/day, n = 18), NKO (0.5 g/kg/day, n = 18), Vit B12 (1 mg/kg/day, n = 18) and IMP (5 mg/kg/day, n = 18). FO:Fish oil; NKO: Neptune Krill Oil; IMP: Imipramine; CUS: Chronic unpredictable stress. Vehicle = 7% Tween 80 in normal saline.

The animal behaviours changes, the body weight and the *post-mortem* brain weight were monitored once a week and the rate of mortality was recorded in a period of 14 days after finishing the tests. Figure 7 depicts a schematic of design used to study anti-depression and anti-anxiety effect of the treatment in this study.

### Forced swimming test (FST); pre-test, test, retest 1

The test was carried out on two successive days after the last stress period, following the method suggested by Szkutnik-Fiedler *et al*.^41^, with modification for application in mice on three different occasions (Pre-test, test and repeated test)^42,43^. On the first day which is called pre-test, all groups of animals (n = 18, in each group) were put individually into the swim cylinders full of tap water (17 cm depth, t = 25 ± 3 °C) to be forced to swim for 15 min of swimming, and then taken out from the water, dried and turned back to their home cages. On the following day, the mice were re-exposed to the forced swimming for another 15 minutes. The score of the immobility (remaining floating in the water) and swimming was recorded when large forepaw movements displaced the body around the cylinder, more than necessary to merely keep the head above the water. Also, climbing was registered when vigorous movements with forepaws in and out of the water, usually directed against the wall of the tank, were observed. For determination the behavioural distribution during the test and retests, all behavioural counts of FST were also recorded minute-by-minute. The behavioural categories were considered distinct, i.e. separated in time for each 2 min interval in the 6 minutes. The behavioural analyses were performed by an experimenter blind to the treatment.

### Repeated FST

Repeated FST is suitable to detect short and long term effects of selective serotonin reuptake inhibitors, or other antidepressant treatment tachyphylaxis, thus reducing the number of animals used in the screenings of this type of compounds. Also, it is a safe experiment to make sure that stress were properly induced and continued in mice by comparison the behaviours of control and stressed groups, therefore, the results of drugs can be more reliable^44^. After 2 days of FST, the animals were again deprived of the food and water for the 4 hrs before conducting the repeated FST and the aforementioned procedure was repeated. The test, retest 1 were videotaped using an infrared video camera set up on 70 cm lateral to the transparent tank, to record subsequent analysis of latency (time elapsed between placing the animal in the tank and the first bout of each behaviour observed), frequency (number of bouts in a 6-min period), and duration (summary of the time spent in all bouts of swimming, climbing, diving, and immobility in a 6-min period).

### Tail suspension test (TST)

All the groups (n = 18 in each group) were transported from the housing room to the testing area in their own cages and allowed to acclimate to their new environment for 1 hr before the test. The test ongoing by suspending the mice on the edge of a holder 50 cm above a table top by adhesive tape placed around 1 cm from the tip of the tail. The duration of the immobility was recorded during a 6 min period and in the last 4 minutes with a video camera and scored by a blinded experimenter, after which, the animals were then returned back to their cages. The mice were immobile when they hanged inactively and were totally unmoving for at least 1 min. A reduction in the length of the immobility time was considered as antidepressant action. In the TST, the total period of immobility was encouraged by the tail suspension measured conferring to the method described by Steru *et al*.^45^.

### Tissue preparation

The pulled out hippocampi of all the groups (n = 18 in each group) were homogenized (1:10 w/v) in the HEPES buffer (20 mM, pH 7.0). The homogenate tissues were centrifuged at 16,000 × g, at 4 °C for 20 min and the supernatants were used for measuring the enzymatic activities and for the quantification of ROS. The total protein was extracted and the amounts were measured by the method described by Lowry *et al*.^46^, using bovine serum albumin as the standard and the same concentration of the extracted protein was used.

### Assessment of brain antioxidant markers

#### Glutathione peroxidase (GPx)

The hippocampal GPx activity was measured through a nicotinamide adenine dinucleotide phosphate (NADPH) decrease assay succeeding the protocol developed by Wendel (1981)^47^. The tissue supernatant (around 200 μg protein) was added to a reaction mixture containing GSH, GR and NADPH in the phosphate buffer (pH 7.4). The reaction was started by adding tert-butyl hydro-peroxide, and the absorbance reduction at 340 nm was measured at 37°. The results were expressed as molar elimination quantity for NADPH of 6.22 × 10^3^ M^−1^ cm^−1^. GPx activity was expressed as nmol NADPH oxidized/min/mg protein and the pure GPX was used as control.

#### Catalase (CAT)

CAT activity was determined to the protocol suggested by Khan *et al*.^48^. The CAT reaction solution consists of 625 μl of 50 mM of potassium phosphate buffer (pH 5), 100 μl of 5.9 mM H_2_O_2_ and 35 μl enzyme extract. Change in the absorbance of the reaction solution was recorded at 240 nm after 1 min. An absorbance change of 0.01 as units/min denotes one unit of catalase activity and the pure CAT was used as control.

#### Superoxide dismutase (SOD)

The SOD activity was assayed spectrophotometrically as described by Misra and Fridovich^49^. This method is built on the capacity of SOD to prevent the autoxidation of the adrenaline to adreno-chrome which was noticed by changing the colour solution at 480 nm. One element of the enzyme was defined as the total of enzyme required to inhibit the amount of the epinephrine autoxidation by 50%. The SOD enzymatic activity was conveyed as units U/mg protein and the pure SOD was used as control.

#### The glutathione (GSH)

The glutathione (GSH) levels were measured as the non-protein thiols (NPSH), based on the protocol advanced by Misra and Fridovich^49^. This procedure calculates only the reduced form of glutathione, which is accountable for the antioxidant properties of this peptide. In addition, a small percentage (around 5%) of other low-molecular weight thiols was similarly measured. Concisely, the hippocampal homogenates (fresh specimen) were precipitated in the 10% cooled tri-chloro-acetic acid and centrifuged at 5000 × g for 10 min, and the supernatant was incubated with the DTNB (5, 5′-dithio-bis(2-nitrobenzoic acid)) in a 1 M phosphate buffer, (pH 7.0) and the absorbance was measured at 412 nm. A standard curve of the reduced glutathione was used to analyse GSH points, which was expressed as nmol of NPSH/mg protein and the pure GSH was used as control.

### Assessment of oxidative stress markers

#### Estimation of malondialdehyde (MDA)

MDA is one of lipid peroxidation product that can be used as a marker for oxidative stress. The assay was carried out following the protocol introduced by Ohkawa *et al*.^50^. The reaction mixture in a total volume of 1.0 ml contained 0.58 ml phosphate buffer (0.1 mol; pH 7.4), 0.2 ml homogenate sample, 0.2 ml ascorbic acid (100 mmol), and 0.02 ml ferric chloride (100 mmol). The reaction mixture was incubated at 37 °C in a shaking water bath for 1 h and was stopped by addition of 1.0 ml 10% trichloroacetic acid. After addition of 1.0 ml 0.67% thiobarbituric acid, all the tubes were placed in hot water bath for 20 min and then placed in an ice bath before centrifuging at 2500 × g for 10 min. The amount of TBARS formed in each of the samples was assessed by measuring optical density of the supernatant at 535 nm, using a spectrophotometer against a reagent blank. The results were expressed as nmol TBARS/min/ mg tissue at 37 °C using a molar extinction coefficient of 1.56 × 105 M^−1^ cm^−1^.

#### Hydrogen peroxide (H_2_O_2_) assay

A reaction mixture, containing 500 μl of 0.05 M phosphate buffer (pH 7), 100 μl of homogenate was added into the mixture containing 100 μl of 0.28 nM phenol red solution, and 250 μl of 5.5 nM dextrose and horse radish peroxidase (8.5 units) and then incubated at room temperature for 60 min. 100 μl of NaOH (10 N) was added to halt the reaction. After centrifuging for 5–10 min (800 × g), the absorbance of the supernatant was calculated at 610 nm. Production of H_2_O_2_ was measured as nm H_2_O_2_/mg tissue by employing the standard curve of phenol red oxidized by H_2_O_2_^51^.

### Statistical analysis

Data were expressed as the mean ± standard error of the mean for the indicated analyses. Levene’s test in advanced was used to make sure the homogeneity of variance and then two way ANOVA analysis of variance (ANOVA) was used for analysing the behavioural data. Following the analyses of variance, Dunnett’s post hoc tests were used. Also, Pearson correlation was used to measuring linearly relation of the two variables. For all analyses, *p* value of < 0.05 (*/^#^/^ǂ^), <0.01 (**/^##^/^ǂǂ^), and <0.001 (***/^###^/^ǂǂǂ^) were considered statistically significant.
