# Author Correction: Antidepressant–like effects of fish, krill oils and Vit B12 against exposure to stress environment in mice models: current status and pilot study

**DOI:** 10.1038/s41598-020-70338-x

**Published:** 2020-08-11

**Authors:** Parastoo Mojtahed Zadeh-Ardabili, Sima Kianpour Rad, Soheila Kianpour Rad, Abolfazl Movafagh

**Affiliations:** 1 Physiology Department, School of Medicine, Mashhad University of Medical Science, Mashhad, Iran; 2 Molecular Medicine Department, Faculty of Medicine, University of Malaya, 50603 Kuala Lumpur, Malaysia; 3 Faculty of Medicine, Qazvin University of Medical Science, Qazvin, Iran; 4 Department of Medical Genetics, School of Medicine, Shahid Beheshti University of Medical Sciences, Tehran, Iran

Correction to: *Scientific Reports* 10.1038/s41598-019-56360-8, published online 27 December 2019

In the original version of this Article, Parastoo Mojtahed Zadeh-Ardabili and Sima Kianpour Rad were omitted as equally contributing first authors. Therefore, the below equal contribution statement has been added:

“These authors contributed equally: Parastoo Mojtahed Zadeh-Ardabili and Sima Kianpour Rad.”

In addition, the author contribution statement has been updated from:

Designing of experiments: Abolfazl Movafagh, Parastoo Mojtahed Zadeh-Ardabili and Sima Kianpour Rad; Consultation: Abolfazl Movafagh; Experimental works: Parastoo Mojtahed Zadeh-Ardabili and Sima Kianpour Rad; Acquisition of data: Sima Kianpour Rad; Analysis and interpretation of data: Sima Kianpour Rad; Drafting of manuscript: Parastoo Mojtahed Zadeh-Ardabili, Sima Kianpour Rad and Soheila Kianpour Rad; Critical revision: Sima Kianpour Rad and Abolfazl Movafagh. All authors contributed toward data analysis, drafting and critically revising the paper, and agree to be accountable for all aspects of the work.

to:

Parastoo Mojtahed Zadeh-Ardabili and Sima Kianpour are considered as first authors in this publication. Designing of experiments: Abolfazl Movafagh, Parastoo Mojtahed Zadeh-Ardabili and Sima Kianpour Rad; Consultation: Abolfazl Movafagh; Experimental works: Parastoo Mojtahed Zadeh-Ardabili and Sima Kianpour Rad; Acquisition of data: Sima Kianpour Rad; Analysis and interpretation of data: Sima Kianpour Rad; Drafting of manuscript: Parastoo Mojtahed Zadeh-Ardabili, Sima Kianpour Rad and Soheila Kianpour Rad; Critical revision: Sima Kianpour Rad and Abolfazl Movafagh. All authors contributed toward data analysis, drafting and critically revising the paper, and agree to be accountable for all aspects of the work.

These errors have now been corrected in the PDF and HTML versions of the paper.

